# Hypodermic Use of Morphia

**Published:** 1879-09-20

**Authors:** B. Greenley

**Affiliations:** Kentucky


### *THE HYPODERMIC USE OF MORPHIA AS A SUPPORTER OF THE HEART'S ACTION.*

BY B. GREENLEY, M.D., OF KENTUCKY.

My object in writing this article is to draw the attention of the profession to the use of morphia hypodermically, in cases which are almost hopeless from loss of blood, or bordering on collapse from exhaustion in attacks of cholera, or severe attacks of vomiting and purging, pernicious chills, etc., and to illustrate its effects by reporting a few cases.

*Case* 1.—Mr. M——, aged 50 years, in delicate health, was taken with diarrhœa and vomiting in the night, the result of drinking a quantity of sweet cider in the evening before retiring to bed. The family, not considering him dangerously sick, did not send for me until late next morning; it being nine o'clock when I arrived at the house. I found him in a state of collapse, but sensible. He was pulseless, very restless; skin purple and cold; still vomiting and purging.

The first thing I did was to inject ⅓ of a grain of morphia under the skin in the region of his heart, as there was no circulation in the extremities. In twenty minutes he expressed himself as feeling greatly relieved. The restlessness subsided, the vomiting and purging ceased, and the skin resumed to some extent its natural color; the pulse could also, in half an hour, be slightly felt at the wrist. In fact, in an hour from the time the morphia was introduced, a most wonderful change in his general condition was wrought. He expresses himself as believing he would get well, and I began to have some hope even, although just before looked upon him as dying.

I now endeavored to maintain the partial reaction thus effected by dry heat applied by means of hot rocks, etc., together with stimulants, both by mouth and injection per anum. But the lost vitality was so great that by the evening of the next day he expired. I have no doubt, had I seen him a few hours earlier, with the same treatment he would have recovered.

*Case* 2.—Mrs. B——, was confined with her 5th child; was subject to hemorrhages on such occasions. I was sent for in haste, the messenger stating she was dying from loss of blood. When I arrived I found her pulseless, cold and perfectly blanched; great nausea and inclination to vomit. The child had been born several hours, and afterbirth still retained. Upon examination found hour-glass contraction and hemorrhage checked.

I gave her ½ grain morphia hypodermically; gradually overcame hour-glass contraction and delivered the placenta. This was in the morning about 10 o'clock, and by giving her iced milk-punch, etc., reaction came on in the afternoon, and she recovered.

*Case* 3.—A child fourteen months old had a chill, and when reaction came on convulsions ensued. When I arrived it had been in continued spasms for over two hours; one side had ceased to jerk. It was pulseless; head thrown back; heart palpitating; panting for breath; eyes turned up and pupils dilated to the margin of the cornea.

I remarked to the mother that the baby was dying, but that I would try an experiment on it; that if it did no good it could do no harm, as the child was, in my estimation, dying.

I injected a small portion of morphia over its heart and waited results. In half an hour I noticed its pupils began to contract, and at the expiration of an hour the spasms ceased, the child went to sleep, and I could now feel the pulse at the wrist. I remained an hour and a half longer when the little patient awoke, recognized its mother and nursed. With the use of quinine to protect it against the recurrence of another chill the patient was well in two days.

*Case* 4.—Was called hurriedly at 2 o'clock at night to see an elderly gentleman who had diarrhœa during the day, and was taken with vomiting in the evening, and cramps coming on in the night. I found him completely prostrated, unable to exercise any control over his bowels; vomiting and purging in bed. His voice had become so changed and weak, you could hardly understand what he said. His legs were greatly cramped; his discharges were of the rice-water character and very profuse.

As soon as I could I injected ½ grain morphia hypodermically, and in half an hour he was comfortable; the vomiting, diarrhœa and cramps having all ceased. By the aid of milk-punch, etc., he was up on the second day.

*Case* 5.—Was called in haste to see Mr. L——, aged about 70 years. He was taken with diarrhœa, succeeded by vomiting and cramps. I found him very much prostrated, and his voice greatly changed and weak.

I gave him ½ grain morphia in the skin, which stopped the vomiting and purging as well as pain, and in half an hour he expressed himself as being entirely relieved, and went to sleep.

These last two cases assimilated cholera very much, and had that disease been prevalent in the country, I should have so pronounced it.

I could report more cases of a similar character, but these will sufficiently demonstrate the object in view.

In all cases of nausea and vomiting, either with or without diarrhœa, the use of morphia in this way will prove beneficial. In cholera-morbus it acts like a charm, relieving all the symptoms in half an hour.

I believe, from the wonderful effects of morphia as a supporter of the heart's action in the cases above reported, that it would prove a reliable remedy in Asiatic cholera, especially when given before complete collapse ensued. Even in some cases of cholera-infantum I should not hesitate to administer it with the hope of saving life.

